# GLP-1 (Glucagon-Like Peptide-1) and GIP (Glucose-Dependent Insulinotropic Polypeptide)/GLP-1 Receptor Agonists for Antipsychotic-Induced Weight Gain: A Narrative Review

**DOI:** 10.7759/cureus.114435

**Published:** 2026-08-12

**Authors:** Agnieszka Buczkowska, Michal Glinski, Julia Danieluk, Natalia Adamaszek, Urszula Kacprzak, Dominika Dolega-Mostowska, Laura Stochaj, Michalina Flejszman, Justyna Wojcik, Julia Sochowska

**Affiliations:** 1 Department of Endocrinology, Metabolism and Internal Medicine, Uniwersytecki Szpital Kliniczny w Poznaniu, Poznań, POL; 2 Department of Internal Medicine, Uniwersytecki Szpital Kliniczny w Poznaniu, Poznań, POL; 3 Department of Medicine, Medical University of Lodz, Łódź, POL; 4 Department of Internal Medicine, Diabetology, Cardiology and Nephrology, Provincial Hospital in Poznań, Poznań, POL; 5 Department of Medicine, University of Life Sciences in Poznań, Poznań, POL; 6 Department of Hypertension, Internal Medicine and Metabolic Disorders, Uniwersytecki Szpital Kliniczny w Poznaniu, Poznań, POL; 7 Department of Pulmonology, Allergology and Pulmonary Oncology, Uniwersytecki Szpital Kliniczny w Poznaniu, Poznań, POL

**Keywords:** antipsychotic-induced weight gain, glp-1 receptor agonists, second-generation antipsychotics, semaglutide, severe mental illness, tirzepatide

## Abstract

The clinical management of severe mental illness faces a profound longevity crisis, as second-generation antipsychotics, particularly clozapine and olanzapine, trigger severe weight gain and cardiometabolic complications that reduce life expectancy by 10 to 20 years. Traditional lifestyle modifications and metformin frequently fail because they do not address the hypothalamic disruption and altered reward signaling caused by these medications. This narrative review synthesizes evidence on advanced incretin-based therapies as a novel counter-regulatory strategy. Glucagon-like peptide-1 receptor agonists, specifically semaglutide, have established a robust benchmark, demonstrating significant weight reduction and cardiovascular risk mitigation in psychiatric cohorts without compromising mental stability. Furthermore, the dual-receptor agonist tirzepatide represents a major paradigm shift, leveraging dual-receptor synergy to provide superior weight loss, optimize adipose tissue function, and reduce hepatic steatosis. Although large-scale randomized controlled trials for tirzepatide in severe mental illness are still limited, preliminary data underscore its extensive role as a comprehensive metabolic stabilizer. Ultimately, closing the mortality gap in psychiatry requires a transition from reactive rescue models toward early, proactive metabolic protection at the onset of antipsychotic therapy.

## Introduction and background

The management of severe mental illness (SMI) currently confronts a systemic crisis of longevity and quality of life. It remains an enduring clinical tragedy that patients diagnosed with schizophrenia and bipolar disorder suffer from a mean life expectancy reduction of 10 to 20 years compared to the general population [1-3]. This mortality gap is not primarily a result of the psychiatric pathologies themselves but is driven by non-communicable cardiometabolic sequelae, with cardiovascular disease (CVD) persisting as the leading cause of death in this cohort [2,4-6]. Standardized mortality ratios for SMI populations indicate that death rates from CVD often represent more than double the rates observed in the general population, a disparity exacerbated by the increased prevalence of obesity, metabolic syndrome, and type 2 diabetes (T2DM) [1,6,7].

A major contributing factor to this metabolic crisis is the clinical utilization of second-generation antipsychotics (SGAs). While high-potency agents like clozapine and olanzapine remain the gold standard for managing treatment-resistant symptoms and preventing relapse, they simultaneously confer the highest liability for clinically significant weight gain and profound metabolic disruption [1,7,8]. Recent literature indicates that GLP-1 receptor agonists (GLP-1 RAs) have emerged as a promising therapeutic approach for mitigating antipsychotic-induced metabolic disturbances and may represent a future standard option pending further high-quality evidence [9-12].

Traditional behavioral and pharmacological interventions are frequently insufficient because they fail to address the unique neurobiological barriers to dieting in patients with SMI. Through their potent antagonism of dopamine D2 receptors, SGAs blunt the mesolimbic reward system [8,13-16]. As a result of dopaminergic D2 receptor antagonism, patients may experience anhedonia and exhibit an increased preference for highly palatable foods, potentially as a compensatory behavioral response [8,13,17,18]. This "craving drive" makes willpower-based dieting and traditional lifestyle modifications physiologically unattainable for many, leading to the high attrition rates and modest results, often a weight reduction of less than 3%, observed in clinical trials of these interventions [1,3,16,19,20].

Given these systemic and molecular failures, the shift toward poly-incretin agonism, specifically the dual glucose-dependent insulinotropic polypeptide (GIP) and GLP-1 RA tirzepatide, represents a significant paradigm shift [11,21]. While GLP-1 RAs have established a new benchmark for weight management in psychiatric populations, tirzepatide offers a potentially enhanced therapeutic effect due to its dual-receptor mechanism and synergistic metabolic actions [11,21,22]. This narrative review synthesizes the current literature, tracing the clinical evolution from GLP-1 mono-agonists to the potent metabolic outcomes of GIP/GLP-1 agonist in the effort to close the mortality gap and restore metabolic equilibrium in patients with severe mental illness.

## Review

Methods

A narrative review was conducted based on a literature search performed using PubMed. The review focused on the pathophysiological mechanisms underlying second-generation antipsychotic (SGA)-induced metabolic dysfunction and the therapeutic potential of glucagon-like peptide-1 receptor agonists (GLP-1 RAs) and the dual GIP/GLP-1 receptor agonist tirzepatide in the management of antipsychotic-induced weight gain and metabolic disturbances. Additional studies addressing the metabolic effects and safety profile of antipsychotic medications were also included. The literature search was performed using the following keywords: “antipsychotic-induced weight gain,” “clozapine,” “olanzapine,” “liraglutide,” “semaglutide,” “tirzepatide,” “GLP-1 receptor agonists,” and “dual GIP/GLP-1 receptor agonist.” Preference was given to randomized controlled trials, systematic reviews, and meta-analyses. Relevant observational studies, case reports, case series, and clinical trial protocols were also included when they provided valuable evidence on the topic. The reviewed literature comprised publications published between 2017 and 2026, allowing the inclusion of both the earliest clinical evidence and the most recent advances in this field.

Pathophysiological mechanisms of antipsychotic-induced metabolic chaos

The metabolic collapse associated with SGAs is not merely a side effect of increased caloric intake but represents a fundamental and multifaceted reorganization of both central energy sensing and peripheral metabolic flux [8,12]. This section delineates the molecular transition from SGA-induced metabolic disruption across the gut-brain-adipose axis to the pathological state that requires intensive therapeutic countermeasures.

Central disruption: receptor overdrive and hypothalamic missignaling

Receptor-Level Overdrive and Orexigenic Signaling

The primary driver of antipsychotic-induced weight gain (AIWG) is a profound disruption of the hypothalamic "nutrient sensing" apparatus [8,23-25]. High-potency SGAs, most notably clozapine and olanzapine, exhibit high-affinity blockade of histamine H1 and serotonin 5-hydroxytryptamine (serotonin) receptor 2C (5-HT2C) receptors [2,12,13]. Histaminergic neurons originating in the posterior hypothalamus project to satiety centers; H1 receptor antagonism by SGAs removes this essential satiety signal, triggering a state of extreme hyperphagia and a relentless orexigenic drive (Appendix 1) [8,13,14].

Simultaneously, SGAs function as potent 5-HT2C inverse agonists [12,14]. Because 5-HT2C receptors are integral to the regulation of energy homeostasis, working in synergy with melanocortin pathways, their blockade disrupts the "satiety brake," leading to significant increases in meal size and frequency [2,12,14]. This receptor-level overdrive shifts the patient into a chronic proanabolic state characterized by an insatiable craving for highly palatable, energy-dense foods [13,25]. The metabolic toxicity of SGAs is further amplified by the activation of 5′-adenosine monophosphate-activated protein kinase (AMPK) within the hypothalamic arcuate and ventromedial nuclei [8,25]. Under physiological conditions, hypothalamic AMPK acts as a master energy sensor that is activated during nutrient deprivation to restore energy balance [2,8,13].

However, SGAs, partially through their potent H1 receptor antagonism, induce a supra-physiological activation of hypothalamic AMPK [2,8,13,14]. This biochemical event creates a false intracellular signal of "cellular energy deficit" or "pseudo-fasting" [8]. In response, the brain triggers a massive sympathetic outflow to the periphery, which inappropriately augments hepatic glucose production via gluconeogenesis and glycogenolysis, while simultaneously stimulating the secretion of glucagon and suppressing the anorexigenic effects of leptin [8,14]. It is important to clarify, however, that the current molecular understanding of hypothalamic nutrient sensing and the activation of the AMPK-sympathetic axis is largely established through findings from preclinical animal research and in vitro studies, as direct verification within human psychiatric populations remains a significant investigative challenge.

Peripheral sabotage: islet cell dysfunction and adiposopathy

Muscarinic M3 Interference and Islet Cell Dysfunction

Beyond central disruption, SGAs engage in a direct peripheral metabolic sabotage by targeting the pancreatic β-cells (Appendix 2) [2,8,12]. Cholinergic muscarinic M3 receptors are critical regulators of glucose-stimulated insulin secretion [2,8,13]. Clozapine and olanzapine possess high affinity for M3 receptors and selectively impair insulin release by blocking these receptors on the islet surface. This islet cell dysfunction occurs independently of weight gain, as evidenced by rapid-onset hyperglycemia in both animal models and healthy volunteers within days of SGA initiation [2,8,12]. The resulting insulin-to-glucagon imbalance facilitates postprandial glucose excursions and accelerates the transition to T2DM [2,8,12].

Pro-adipogenic effects and lipogenesis

SGA-induced weight gain is synonymous with a transition to pathological adipose tissue function, or adiposopathy [8,26-28]. SGAs profoundly interfere with lipid homeostasis by upregulating sterol-regulatory element-binding proteins (e.g., SREBP-1c), which are master transcriptional regulators of lipogenesis [8]. Chronic exposure to agents like olanzapine stimulates the differentiation of pre-adipocytes into mature adipocytes and promotes adipocyte hypertrophy [2,8]. This pro-adipogenic environment results in excessive visceral fat deposition and a systemic increase in circulating free fatty acids, which further exacerbate insulin resistance in skeletal muscle and the liver [8].

Adiponectin impairment and chronic inflammation

A hallmark of SGA-induced metabolic disruption is the significant impairment of the insulin-sensitizing hormone adiponectin [8,12,14,25,27]. SGAs, clozapine and olanzapine in particular, induce hypoadiponectinemia, which removes a vital anti-inflammatory and insulin-sensitizing signal [12,14]. This hormonal deficit, coupled with the hypertrophy of visceral fat, triggers a low-grade pro-inflammatory state, characterized by elevated levels of pro-inflammatory markers such as tumor necrosis factor alpha (TNF-α) and interleukin-6 (IL-6) [8,12].

The gut-brain axis breakdown: dysbiosis and endogenous incretin deficit

Furthermore, SGAs may induce alterations in gut microbiota composition, shifting the intestinal microbial profile toward obesity-associated characteristics, including changes in the Firmicutes-to-Bacteroidetes (F:B) ratio [9,14]. Chronic exposure to agents such as clozapine and olanzapine has been associated with disturbances in microbial diversity and gut metabolic homeostasis, which may contribute to antipsychotic-induced metabolic complications [8,9]. However, the extent to which these microbiota alterations represent a cause or consequence of metabolic dysfunction remains incompletely understood [8,9].

Gut microbiota disruption may contribute to metabolic abnormalities through modulation of inflammatory pathways and impaired gut-brain communication [8,9]. These changes may promote a chronic low-grade inflammatory state, further exacerbating insulin resistance and cardiometabolic risk in patients receiving SGAs [8]. Importantly, emerging evidence suggests that microbiota alterations may also influence endogenous glucagon-like peptide-1 (GLP-1) signaling from intestinal L-cells [9].

Under physiological conditions, endogenous GLP-1 acts as a key mediator of the gut-brain axis by promoting satiety signaling and maintaining glucose homeostasis [9]. Disruption of this pathway may represent a potential mechanistic link between SGA exposure and metabolic dysfunction, providing a biological rationale for investigating exogenous GLP-1 receptor agonists as a therapeutic strategy in this population [7,9]. Nevertheless, direct causality between microbiota changes and impaired incretin signaling remains unconfirmed, particularly in human psychiatric populations [7].

The incretin revolution: from mono-agonism to poly-incretin synergy

Incretin mimetics, such as GLP-1 RAs, act as potent restorative agents by bypassing the receptor-level blocks established by SGAs [17,26,29,30]. Mechanistically, these agents directly stimulate the anorexigenic pro-opiomelanocortin and cocaine- and amphetamine-regulated transcript neurons while simultaneously inhibiting the orexigenic neuropeptide Y and agouti-related protein neurons within the arcuate nucleus [1,14,17,31]. By restoring this vital satiety brake, GLP-1 RAs counteract SGA-induced hyperphagia, normalize food-related reward signaling in the mesolimbic pathway, and delay gastric emptying, thereby smoothing postprandial glucose excursions [3,7,26,30,32-33].

Tirzepatide, a dual GIP and GLP-1 receptor agonist, represents an enhanced therapeutic modality due to its synergistic, imbalanced agonism that structurally favors GIP receptor activity [11,21,23]. While the GLP-1 component primarily drives central satiety, GIP receptor engagement provides unique benefits in peripheral therapeutic targets [8,11,21,23]. Specifically, GIP signaling improves white adipose tissue function by enhancing lipid buffering, increasing the efficiency of fat storage in white adipose tissue and reducing ectopic fat deposition in the liver and muscle that drives insulin resistance [8,21,23,32]. Recent preclinical data suggest that tirzepatide robustly shifts substrate preference toward fat utilization by enhancing lipolytic and thermogenic capacity in adipose tissues via sympathetic nervous system innervation [24]. This dual action allows for high-dose weight-loss efficacy, exceeding 20%, while potentially mitigating the dose-limiting gastrointestinal distress like nausea/vomiting associated with pure GLP-1 agonists [3,21,24,32,34,35].

Beyond metabolic restoration, both GLP-1 and GIP receptors are widely expressed in the human brain, including cortical areas associated with memory, emotion, and cognitive processing [18,20,26,36]. These agents exert potent neuroprotective effects by stimulating the production of brain-derived neurotrophic factor, enhancing synaptic plasticity, and reducing oxidative stress [1,3,11,26,28,30]. The ability of incretin mimetics to suppress microglial activation and reduce neuroinflammation offers a potential dual benefit: restoring metabolic equilibrium while simultaneously protecting against the cognitive decline and accelerated brain aging often observed in schizophrenia and bipolar cohorts [3,11,15,20,26,30].

Clinical evidence and weight-loss outcomes

GLP-1 RAs: Establishing the Clinical Benchmark

The clinical management of AIWG has historically relied upon modest pharmacological interventions, with metformin serving as the traditional first-line choice despite a placebo-subtracted weight loss rarely exceeding 3% (approximately 2.5 to 3.3 kg) [1,3,16,19,20]. The arrival of GLP-1 RAs has significantly altered this landscape, providing a physiological counter-regulatory effect to the orexigenic overdrive induced by SGAs [7,10,13]. Emerging clinical data emphasize that the therapeutic response within SMI populations remains highly variable, suggesting that the potent receptor-binding profiles of high-potency antipsychotics may partially interfere with the central weight-lowering mechanisms of incretin mimetics [1,10].

The liraglutide era: foundational evidence

Liraglutide, a long-acting GLP-1 analog, provided the first robust evidence for the efficacy of incretin mimetics in the psychiatric population [7,17]. Foundational randomized controlled trials, most notably the work of Larsen et al. [37], targeted the highest-risk cohorts: patients on clozapine or olanzapine with pre-existing obesity and prediabetes. In this 16-week, double-blind trial, liraglutide (titrated to 1.8 mg/day) induced a placebo-subtracted weight loss of 5.3 kg [37]. Beyond weight reduction, the trial demonstrated significant metabolic improvement, with 63.8% of the liraglutide group achieving normal glucose tolerance compared to only 16% in the placebo arm [37].

These findings were corroborated by the study conducted by Whicher et al. [38], which utilized a higher 3.0 mg daily dose over 24 weeks, resulting in a weight reduction of 5.7 ± 7.9 kg and significant improvements in glycated hemoglobin (HbA1c) and waist circumference [38]. However, the 68-week follow-up study by Svensson et al. [39] revealed a critical clinical caveat: while weight loss was largely maintained one year after drug withdrawal, nearly all other metabolic parameters (fasting glucose, lipid profiles, and HbA1c) regressed to baseline values, suggesting that GLP-1 RA therapy may require chronic administration to sustain metabolic equilibrium in the context of ongoing SGA use [39]. Despite these successes, the burden of daily subcutaneous administration remains a significant hurdle for patients with SMI who often contend with cognitive deficits and avolition, leading researchers to seek more potent, once-weekly alternatives [3,39].

Semaglutide: the potency leap and adherence paradigm

Semaglutide represents a second-generation enhanced therapeutic effect in GLP-1 RA therapy, offering both greater receptor affinity and a significantly extended half-life [3]. In the general obesity population, the Semaglutide Treatment Effect in People with obesity (STEP) trials demonstrated substantial efficacy, with STEP 1 showing a 14.9% mean weight loss over 68 weeks [22]. Crucially, the STEP 8 trial directly compared weekly semaglutide (2.4 mg) with daily liraglutide (3.0 mg), finding that semaglutide was nearly three times as effective for weight reduction (15.8% vs. 6.4%) and was associated with fewer treatment discontinuations due to adverse events [3].

In psychiatry, the Clozapine Obesity and Semaglutide Treatment (COaST) trial supports semaglutide as a leading therapeutic option for AIWG [31]. The results demonstrated a 13.88% weight reduction in the semaglutide group, contrasted with a 0.42% increase in the placebo group [31]. Furthermore, 93.3% of participants achieved ≥5% weight loss, a threshold associated with significant cardiovascular risk reduction [3]. From a clinical integration perspective, the transition to once-weekly dosing is a critical factor for adherence in SMI patients [1,3]. By reducing the frequency of injections, semaglutide addresses the neurobiological barriers of treatment-resistant schizophrenia, potentially improving long-term cardiometabolic outcomes without altering clozapine plasma levels or worsening psychiatric stability [31].

Tirzepatide: breaking the monotherapy ceiling

While semaglutide has established a high benchmark, tirzepatide represents the arrival of poly-incretin agonism, a potential shift in therapeutic approach that promises to overcome the reduced metabolic responsiveness frequently encountered in patients treated with high-potency antipsychotics (Appendix 3) [11,21]. Clinical comparisons indicate that tirzepatide provides a more significant dose-dependent weight-loss effect than GLP-1 mono-agonists [21,22]. The Study of Tirzepatide in Participants With Obesity or Overweight for the Treatment of Obesity (SURMOUNT-1) trial [22], involving over 2,500 participants with obesity, demonstrated that the 15 mg weekly dose achieved a mean weight loss of 20.9% (22.5 kg) over 72 weeks [22]. This substantial efficacy was mirrored in the Study of Tirzepatide Once Weekly Compared With Semaglutide Once Weekly in Participants With Type 2 Diabetes (SURPASS-2) trial [21], where tirzepatide proved significantly superior to semaglutide (1 mg) in both glycemic control and body weight reduction [21]. For psychiatric patients who have failed on metformin or liraglutide, this dual-receptor engagement may provide the necessary metabolic stimulus to bypass SGA-induced insulin resistance and leptin signaling deficits [1,11].

Importantly, it should be noted that there is currently a lack of large-scale randomized controlled trials investigating the use of tirzepatide specifically within schizophrenia or bipolar disorder populations [1]. As a result, the existing evidence base for its application in these groups remains preliminary or extrapolated from data established in general metabolic and obesity cohorts [1]. While large-scale RCTs for tirzepatide in schizophrenia are currently in the pipeline, early real-world data and case reports suggest its utility in improving outcomes even in severe cases of AIWG [24,25].

A recent case report by Lim et al. [40] detailed the successful use of tirzepatide in a clozapine-treated patient who had developed severe obesity and metabolic syndrome [40]. Furthermore, analogous data from the general schizophrenia population using the older dual agonist exenatide [24] demonstrated that some patients could lose up to 41.5 kg in 6 months when the incretin system was successfully engaged, suggesting that the ceiling of metabolic restoration in psychiatry may be higher than previously thought [24]. In Asian psychiatric cohorts, a group often more susceptible to metabolic side effects at lower BMI thresholds [25], preliminary evidence from the SURMOUNT trial in China (SURMOUNT-CN) and SURMOUNT trial in Japan (SURMOUNT-J) trials [25] has confirmed that tirzepatide remains safe and highly effective, with up to 96% of participants achieving ≥5% weight reduction [25]. These studies underscore the potential of tirzepatide to act as a potential therapeutic option for patients who exhibit rapid, early weight gain upon the initiation of clozapine or olanzapine [25,40].

The clinical value of tirzepatide extends far beyond simple weight loss; it functions as a comprehensive metabolic stabilizer [11,21]. In a substudy of the SURPASS-3 MRI trial [32], tirzepatide was shown to produce a significant reduction in liver fat content and visceral adipose tissue compared to insulin degludec [32]. This is of paramount importance in SMI, where metabolic-associated steatotic liver disease (MASLD) is highly prevalent due to SGA-induced lipogenesis [8]. Tirzepatide treatment consistently results in the lowering of circulating triglycerides, apolipoprotein C-III, and small dense low-density lipoprotein (LDL) particles, while increasing adiponectin levels, a hormone with potent anti-inflammatory and insulin-sensitizing properties [11,21,33]. Analysis of the SURMOUNT-1 trial [22] showed a net reduction of 6.8 mmHg in systolic blood pressure, a correction largely mediated by the drug’s profound effects on body composition and sympathetic tone [22].

By addressing adiposopathy, hepatic steatosis, and hypertension simultaneously, tirzepatide offers a holistic pharmacological strategy that may contribute to reducing cardiometabolic risk in psychiatric populations [11,34]. Nevertheless, it must be noted that while these agents demonstrate significant cardiometabolic improvements, such outcomes are not yet definitively proven to reduce actual mortality rates within the severe mental illness population [1]. As clinicians move toward a precision psychiatry model, tirzepatide’s ability to restore metabolic equilibrium in the face of ongoing antipsychotic therapy positions it as a promising direction in the pursuit of functional recovery and longevity for individuals with severe mental illness [11,21].

Clinical integration and neuropsychiatric safety

The integration of incretin-based therapies into psychiatric care requires a nuanced balance between metabolic restoration and psychiatric stability [1]. As these agents move toward becoming the standard of care for AIWG, clinicians must navigate both the safety data and the practical barriers to implementation [3]. A critical point of clinical concern has been the potential association between GLP-1 RAs and suicidal ideation, particularly as these agents are centrally active [30]. While some early post-marketing reports raised alarms, recent large-scale real-world analyses have provided significant reassurance [32]. Pharmacovigilance studies analyzing the FDA Adverse Event Reporting System and EudraVigilance found that while reports of suicidal thoughts exist, the reporting odds ratios for GLP-1 RAs are often lower than those for other common metabolic medications like metformin [30]. Crucially, the United States Food and Drug Administration (FDA) and the European Medicines Agency concluded in 2024 that current evidence does not support a causal link between GLP-1 RAs and suicidal behavior [32]. Furthermore, a landmark retrospective cohort study utilizing electronic health records from over 200,000 patients found that semaglutide was associated with a lower risk of suicidal ideation compared to non-GLP-1 RA anti-obesity or anti-diabetic medications [30].

Beyond safety, there is compelling evidence for pro-psychiatric effects. Mendelian randomization studies suggest a causal relationship between genetically proxied GLP-1 receptor activation and a reduced risk of schizophrenia [36]. Mechanistically, these agents exert potent neuroprotective effects by increasing brain-derived neurotrophic factor and reducing neuroinflammation, which may improve cognitive dimensions of SMI that remain unaddressed by standard antipsychotics [3,11].

Somatic adverse effect management and practical barriers

The successful clinical integration of incretin-based therapies in patients with SMI necessitates a proactive and meticulous approach to adverse effect management [11]. Given that SGAs already contribute to a substantial medication burden, the introduction of GLP-1 or GIP/GLP-1 agonists should follow a slow and gradual dose-titration protocol to ensure long-term tolerability and minimize premature treatment discontinuation [21]. For tirzepatide, the standard initiation begins at a sub-therapeutic dose of 2.5 mg once weekly for the first four weeks [21]. Clinical evidence suggests that dose escalation should proceed in 2.5 mg increments every four weeks, moving to 5 mg, 7.5 mg, 10 mg, 12.5 mg, and finally 15 mg [22]. This deliberate pace allows for physiological adaptation of the gastrointestinal tract (GI) and has been shown to significantly reduce the severity of treatment-emergent symptoms [21].

In psychiatric cohorts, where patients may be more sensitive to somatic changes, clinicians are encouraged to remain at a specific dose level for an additional four weeks if mild GI distress persists, rather than adhering to a rigid escalation schedule [1]. The primary clinical challenge for psychiatric practitioners remains the management of GI side effects, specifically nausea (reported in 31%-41% of users), vomiting (17%-25%), and diarrhea [21]. Clinicians should be reassured that these symptoms are typically mild to moderate and transient, predominantly occurring during the initial weeks of treatment or immediately following a dose increase, and generally subside over time as metabolic adaptation occurs [24]. To mitigate these effects, patients should be counseled on dietary modifications, such as consuming smaller, more frequent meals, reducing intake of high-fat foods, and prioritizing hydration to avoid volume depletion, which is particularly critical for patients on concurrent lithium or diuretics [1]. If nausea persists, the use of a "dose-hold" strategy or a temporary reduction to the previously tolerated dose is often sufficient to maintain the therapeutic alliance without compromising psychiatric stability [3].

Regarding rare but severe risks, recent 2024 and 2025 meta-analyses have provided crucial clarity [32]. While early concerns linked GLP-1 RAs to an increased risk of acute pancreatitis, the current consensus from large-scale real-world data and network meta-analyses indicates no significant difference in the risk of pancreatitis compared to placebo or other anti-diabetic medications in patients with obesity or type 2 diabetes [32]. However, gallbladder-related disorders, such as cholelithiasis and acute cholecystitis, have been associated with both rapid weight loss and the pharmacological effects of incretin mimetics, with liraglutide and semaglutide showing a higher reporting probability than dual agonists [35].

Practical integration, economic barriers, and preventative timing

The therapeutic potential of these agents is frequently limited by cost and accessibility [1]. GLP-1 RAs are high-cost medications, and public insurance coverage is often restricted to patients with a primary diagnosis of T2DM, effectively excluding many SMI patients who have not yet reached that threshold [6]. Given that up to 90% of individuals with schizophrenia are unemployed, private insurance is rarely a viable alternative [1].

Furthermore, clinical practice is often reactive rather than proactive. Experts argue that metabolic intervention should move from a rescue therapy model to a preventative strategy [7]. Waiting for massive weight gain to occur before initiating incretin therapy allows for the accumulation of irreversible cardiovascular damage [2]. Initiating agents like semaglutide or tirzepatide early, ideally at the start of high-potency antipsychotic therapy, may prevent the metabolic cascade and improve overall treatment adherence by removing the distressing side effect of weight gain [7].

Future directions and limitations

The landscape of metabolic management in psychiatry is rapidly evolving from simple weight suppression to the restoration of complex neuro-metabolic equilibrium. The next frontier in restoring metabolic equilibrium involves several key developments that could reshape current treatment paradigms. First, the emergence of oral formulations, such as oral semaglutide and other oral small-molecule GLP-1 receptor agonists, may directly address "injection fatigue" and substantially improve long-term adherence in SMI populations [1,14,26]. Furthermore, the therapeutic potential is being expanded through triple agonism; investigational agents like retatrutide (GLP-1/GIP/glucagon) promise even greater weight-loss potency by adding a third metabolic pathway to increase energy expenditure [30,32]. Finally, accumulating evidence suggests that AIWG must be treated as a chronic condition, given that weight loss is frequently regained after incretin discontinuation [26]. Consequently, future research must shift toward establishing long-term maintenance protocols tailored for patients undergoing lifelong antipsychotic therapy.

Despite the therapeutic promise of incretin mimetics, several limitations in the selected literature must be acknowledged. The primary limitation remains the stark lack of large-scale, long-term randomized controlled trials investigating the use of the newest generation of agents, specifically tirzepatide, within schizophrenia or bipolar disorder populations [1]. Consequently, much of the high-potency data regarding dual receptor agonism must be extrapolated from general obesity and type 2 diabetes cohorts, which do not completely mirror the unique neurobiological and behavioral challenges faced by patients with SMI.

Furthermore, the specific clinical relationships within the gut-brain axis, such as the exact causal connection between antipsychotic-induced microbiota alterations and the subsequent decline in endogenous GLP-1 secretion, remain predominantly associative and require more direct mechanistic verification in human psychiatric subjects [28]. Lastly, while current clinical evidence confirms substantial metabolic correction, existing studies are not yet longitudinal enough to definitively prove that these metabolic interventions translate to a measurable reduction in long-term mortality rates or a full closing of the 10- to 20-year life expectancy gap within the SMI population [1,6].

## Conclusions

The metabolic toxicity of SGAs significantly contributes to a 10- to 20-year reduction in life expectancy in patients with SMI. These disorders are driven by hypothalamic energy-sensing disruption and peripheral adiposopathy, creating a state of chronic metabolic pseudo-fasting that resists traditional interventions. In response, GLP-1 RAs provide a robust physiological counterweight to antipsychotic-induced weight gain. Agents like semaglutide demonstrate significant weight reduction and cardiovascular improvement without compromising psychiatric stability. Building upon this, tirzepatide leverages dual-receptor synergy to bypass metabolic resistance as a comprehensive stabilizer. However, its application remains constrained by a lack of large-scale RCTs in schizophrenia and bipolar disorder populations. Ultimately, bridging the mortality gap necessitates a shift toward proactive metabolic intervention at the onset of antipsychotic therapy. Integrating advanced metabolic protection into the standard of care can restore neuro-metabolic equilibrium and significantly improve longevity for individuals living with SMI.

## Figures and Tables

**Figure 1:**
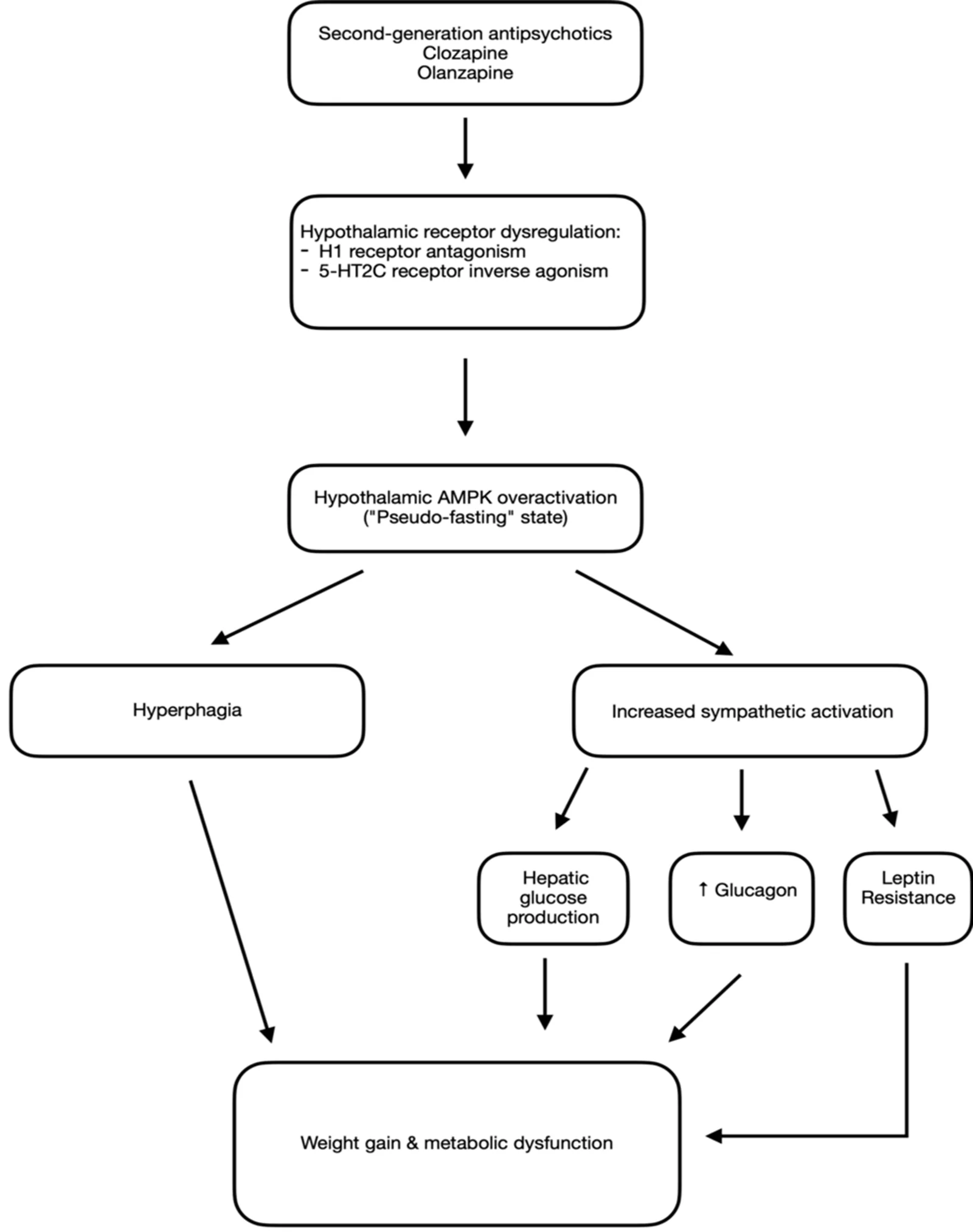
Mechanisms of hypothalamic receptor dysregulation and AMPK activation underlying antipsychotic-induced weight gain. 5-HT2C: 5-hydroxytryptamine (serotonin) receptor 2C; AMPK: 5'-adenosine monophosphate-activated protein kinase; H1, histamine H1 receptor; SGAs, second-generation antipsychotics.

**Figure 2:**
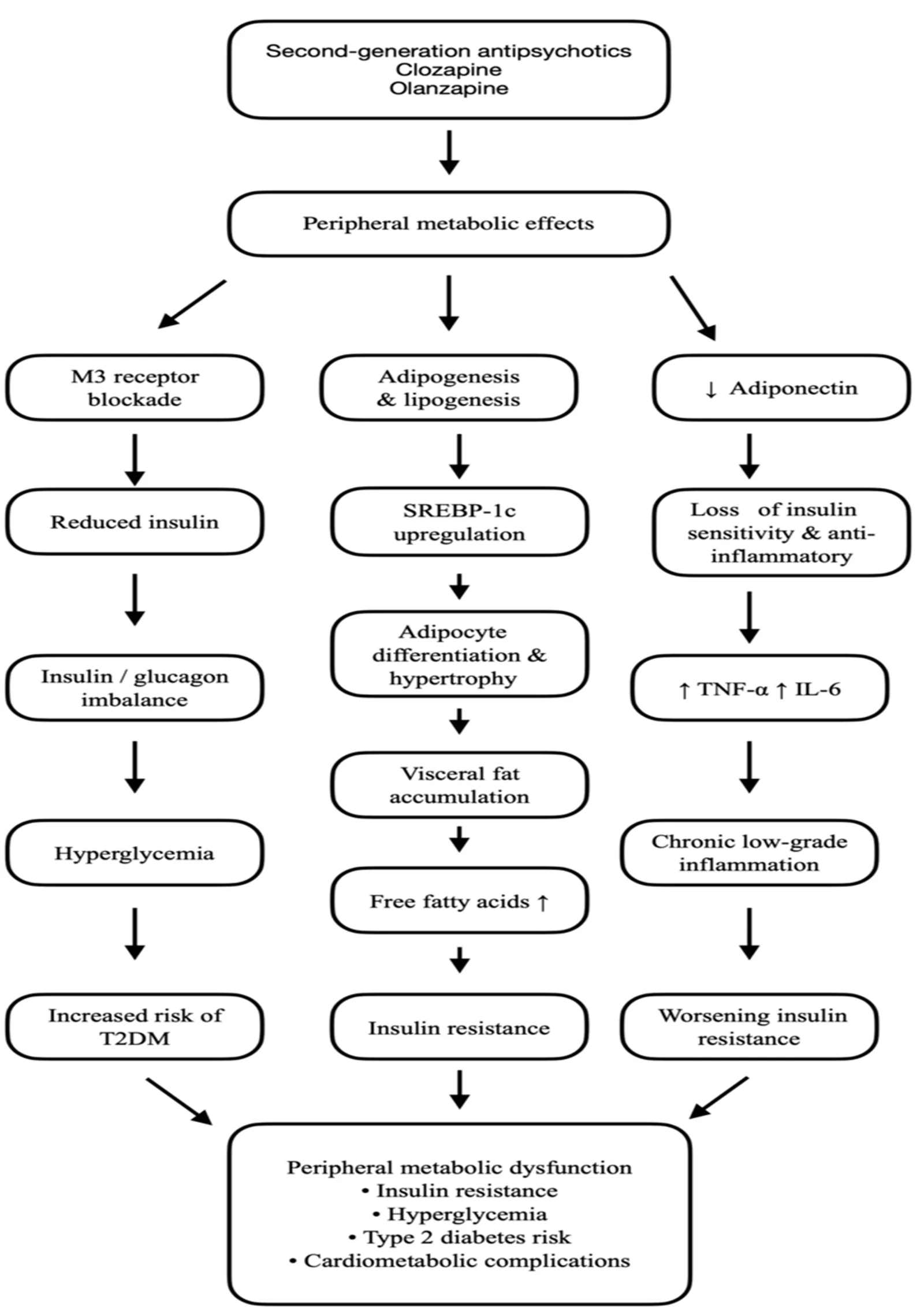
Peripheral mechanisms underlying antipsychotic-induced metabolic dysfunction. FFA, free fatty acids; IL-6, interleukin-6; M3, muscarinic acetylcholine receptor M3; SGAs, second-generation antipsychotics; SREBP-1c, sterol regulatory element-binding protein 1c; T2DM, type 2 diabetes mellitus; TNF-α, tumor necrosis factor alpha.

## Appendices

Appendix 1

Appendix 2 

Appendix 3 

**Table 1:** Comparative analysis of pharmacological interventions for antipsychotic-induced weight gain and metabolic dysfunction in severe mental illness. AIWG, antipsychotic-induced weight gain; ApoC-III, apolipoprotein C-III; BP, blood pressure; HbA1c, glycated hemoglobin; LDL, low- density lipoprotein; RCT, randomized controlled trial; s.c., subcutaneous; SGA, second-generation antipsychotics; SMI, severe mental illness; T2DM, type 2 diabetes mellitus; STEP, Semaglutide Treatment Effect in People with obesity.

Therapy	Dosing	Weight loss	Key metabolic benefits	Current evidence in SMI	Main limitations
Metformin	Oral, daily	~2.5-3.3 kg (≈3% placebo-subtracted) [1,3,16,19,20]	Modest improvement in insulin sensitivity and glycemic control	Established first-line pharmacotherapy for AIWG	Limited weight-loss efficacy
Liraglutide	1.8-3.0 mg, daily s.c.	5.3-5.7 kg [37,38]	Improved glucose tolerance, HbA1c, and waist circumference; metabolic benefits maintained only during treatment [37-39]	Robust RCT evidence in clozapine-/olanzapine-treated patients [37,38]	Daily injections; metabolic benefits diminish after discontinuation [39]
Semaglutide	2.4 mg, weekly s.c.	14.9% (STEP 1) [22]	Sustained weight reduction; high proportion achieving ≥5% weight loss; improved adherence with weekly dosing [3,31]	Promising evidence; dedicated schizophrenia studies emerging	Limited long-term evidence specifically in SMI
Tirzepatide	Up to 15 mg, weekly s.c.	Up to 20.9% (22.5 kg) [22]	Reduced liver fat, visceral adiposity, triglycerides, ApoC-III and small dense LDL; increased adiponectin; reduced systolic BP by 6.8 mmHg [21,22,32,33]	Preliminary psychiatric evidence (case reports and extrapolation from obesity/T2DM trials) [24,25,40]	No large randomized trials in schizophrenia or bipolar disorder
